# Sensorless PV Power Forecasting in Grid-Connected Buildings through Deep Learning

**DOI:** 10.3390/s18082529

**Published:** 2018-08-02

**Authors:** Junseo Son, Yongtae Park, Junu Lee, Hyogon Kim

**Affiliations:** 1 B2B Solution R&D Center, CTO, LG Electronics, 51, Gasan digital 1-ro, Geumcheon-gu, Seoul 08592, Korea; junseo.son@lge.com (J.S.); junu.lee@lge.com (J.L.); 2 Department of Computer Science and Engineering, Korea University, Anam-Dong, Sungbuk-gu, Seoul 02841, Korea; ytpark@korea.ac.kr

**Keywords:** solar power, deep learning, PV power output forecast, on-site meteorological sensors, cost reduction, accuracy

## Abstract

Existing works in photovoltaic (PV) power generation focus on accurately predicting the PV power output on a forecast horizon. As the solar power generation is heavily influenced by meteorological conditions such as solar radiation, the weather forecast is a critical input in the prediction performance. However, the weather forecast is traditionally considered to have coarse granularity, so many are compelled to use on-site meteorological sensors to complement it. However, the approach involving on-site sensors has several issues. First, it incurs the cost in the installation, operation, and management of the sensors. Second, the physical model of the sensor dynamics itself can be a source of forecast errors. Third, it requires an accumulation of sensory data that represent all seasonal variations, which takes time to collect. In this paper, we take an alternative approach to use a relatively large deep neural network (DNN) instead of the on-site sensors to cope with the coarse-grained weather forecast. With historical PV output power data from our grid-connected building with a rooftop PV power generation facility and the publicly available weather forecast history data, we demonstrate that we can train a six-layer feedforward DNN for the day-ahead forecast. It achieves the average mean absolute error (MAE) of 2.9%, comparable to that of the conventional model, but without involing the on-site sensors.

## 1. Introduction

**Figure 1:**
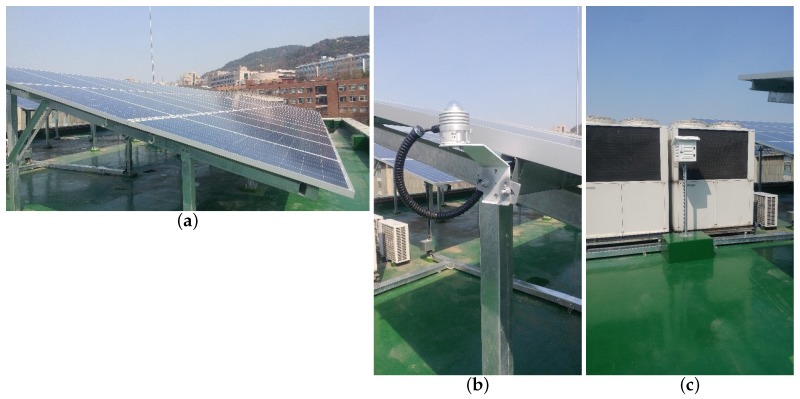
Our solar power system installation site used for the experiments in this paper. (**a**) solar panels (Amorphous Silicon); (**b**) solar irradiance sensor installed near the solar panels; (**c**) temperature and humidity sensors in the white-painted instrument shelter.

Recently, the solar power generation method has been shifting its focus from the Concentrating Solar Power (CSP) system to the grid-connected photovoltaic (PV) power generation [1]. The key aspect of managing the micro-grid environment, such as a grid-connected building, is balancing between the amounts of power generation and the demand. Because the solar power generation is strongly affected by weather conditions, forecasting the generated solar power in the face of the weather changes is a critical component for the management [2]. A difficulty in the forecast is the granularity and quality of the weather forecast used as input to the PV power output forecasting. As the weather forecast is traditionally considered to have coarse granularity, many are compelled to use on-site meteorological sensors to complement it. For such conventional systems, on-site sensors such as irradiance, temperature, and humidity sensors are usually installed together with the solar panels (Figure 1). Typically, these systems use a two-stage approach [3]. First, they model the relation between the regional weather forecast and the precise on-site measurement at the forecasted time. Then, using the precise historical on-site measurement values inferred from the relation and the weather forecast input, they use a PV model (typically implemented in commercial software) to forecast the PV power output.

However, this two-stage approach involving on-site sensors has several issues. First, it incurs the cost in the installation, operation, and management of on-site sensors. Moreover, it incurs the engineering cost to reflect the sensor readings and the solar panel characteristics to the prediction. Second, the physical model of the sensor dynamics itself can be a source of forecast errors. Third, it requires an accumulation of sensory data that represent all seasonal variations, which takes time to collect. Therefore, in this paper, we aim to show the feasibility of an alternative approach that do not depend on the supplementary on-site sensor hardware modules in the PV output power forecast. Specifically, the alternative approach employs a relatively large deep neural network (DNN) to cope with the coarse-grained weather forecast. Indeed, by training the DNN with a year’s worth of the public weather forecast data and the contemporary power generation history from our testbed, we demonstrate that the DNN forecast model produces a higher level of prediction performance in almost all measures.

We consider this problem in the context of the grid-connected building that utilizes the solar power as a supplement to the traditional power provisioning, as shown in Figure 1. With the historical PV output power data from our rooftop PV power generation facility and the weather forecast data for the building location, we train a 6-layer feedforward DNN for the day-ahead forecast. We demonstrate that it achieves the average mean absolute error (MAE) of 2.9%, comparable to that of the conventional model we have used involving the on-site sensors. We believe that the alternative forecast model can simplify the grid-connected building energy management systems (BEMS), making it more cost-effective.

## 2. Related Work

There is very rich literature in solar power forecasting, and an excellent survey of prior work can be found in Inman et al. [4]. Current approaches to predicting the solar power generation are classified into physical, statistical, machine-learning, and hybrid methods [5] that combine any of these methods. The physical methods mathematically model the physical state of the facility, geographical location, meteorological variables at the location and historical data to predict the solar radiation [6,7]. Their accuracy depends on the stability of the weather conditions [8]. The statistical models include regression models such as multiple regressions [9], exponential smoothing [10], auto-regressive moving average (ARMA) [11,12,13], and auto-regressive integrated moving average (ARIMA) [14,15]. The performance of these models is good for short forecast horizons from few minutes to few hours [16,17]. However, they are not flexible to the sudden change of time series due to the fixed parameters. Namely, they do not have good approximation for nonlinear time series or sudden changes [18]. Thus, there have been works that overcome these shortcomings. For instance, Piorno et al. [19] improved upon exponentially weighted moving average (EWMA) by additionally considering the current weather conditions for better accuracy and lower computation overheads. For further improvement, Bergonzini et al. [20] applied the so-called phase displacement regulator (PDR) that utilized a feedback response to reflect the error in the past predictions. In essence, statistical methods are still evolving towards higher sophistication and accuracy while reducing the computation and memory requirements. Machine learning methods are more popular recently, and they range from more conventional techniques such as support vector machine (SVM) [21,22,23] to artificial neural networks (ANN).

As we focus on the artificial neural networks (ANN)-based approach in this paper, we refer the readers to the work for other more traditional approaches, such as physical and statistical models. In particular, we classify existing work into the broad categories of those using on-site sensors and those that do not. First, there are works that use purely the measurement data from on-site sensors. Cococcioni et al. [24] used the on-site irradiation history data for the last 15 days to directly forecast with a one-day horizon using a feedforward neural network. The 15-day data serve as the temporal context for the forecast model. The network has a single hidden layer with 10 neurons. Mandal et al. [25] used wavelet transform (WT) and ANN for one-hour-ahead power output forecasting, using 30-day solar radiation and temperature data collected from on-site. Mellit [26] also used total solar radiation and temperature measured at the site to train an Elman network to predict the next day’s power generation. It showed that the Elman network has a better prediction error than Multi-Layer Perceptron (MLP). Chu et al. [27] used on-site camera and lidar to obtain the cloud cover and wind speed information. The work used the measured data to train the feedforward ANN model, and use it to forecast up to 15 min in the future. Zhu et al. [28] used wavelet decomposition to remove the high-frequency disturbances from the on-site meteorological input values. Then, for each input parameter, the low-frequency wavelet layers were input to a single hidden layer ANN, whose output are combined and wavelet reconstructed. Zhu et al. [29] used the on-site solar irradiance, temperature, and humidity measurements from the most recent 25 days instead of from the whole year (‘scrolling window’) to train and update a 3-layer back propagation (BP) network model. The authors showed that it could improve the quality of the forecast as it could better model the non-stationary climatic changes over the year. The number of neurons in the three hidden layers were 50, 30, and 1, respectively. Yousif et al. [30] used a Self-Organizing Feature Map (SOFM) with one hidden layer to predict daily power output using the solar radiation and ambient temperature data measured at the site. In comparison with these works, our work uses a larger feedforward neural network with multiple hidden layers and a large number of neurons in each hidden layer. We train the DNN with an entire year’s weather forecast only, whose time dependency we do not exploit as some of the aforementioned works did. It is because the weather forecast data we use have the 3-h resolution, and we believe that any dynamic weather condition changes between the forecasts may not be modelled well by the time dependency.

There are also works that combine the on-site measurements with the weather forecast data as input. Chupong et al. [31] used the Elman neural network, a type of Recursive Neural Network (RNN), to forecast the next day power generation using the forecasted clear sky solar radiation (CSRM) and the local weather forecast. The local weather forecast included the max and min temperatures and the cloud cover index. The CSRM needed to be translated to the on-site solar radiation based on the installation parameters of the facility such as the tilting angle of the panel. Leva et al. [32] trained a single hidden layer ANN using the weather forecast and the historical on-site solar irradiance to predict the photovoltaic power output in the 24-h horizon. It claimed that the on-site solar irradiance measurement is important for evaluating the accuracy of the ANN method and the weather forecast. Ramsami et al. [33] also used daily historical on-site measurements of meteorological variables and weather forecast data to train a MLP. Our work sharply contrasts with these works in that we show that the forecast performance with a relatively large DNN is no worse than the model relying on the on-site sensors. In addition, we do not use the irradiance input in our model, so we do not need the installation parameters of the facility.

Finally, there are works that rely only on the weather forecast. These are the most similar works to ours. Chen et al. [3] used the forecast data from online meteorological services for the 24 h PV output forecast. It used a hybrid of self-organized map (SOM) to classify the input variables, and a three-layer radial basis function network (RBFN) for each weather type. It used a single hidden layer, with 5, 10, or 15 neurons. Yona et al. [34] used Fuzzy Theory to predict the solar radiation based on the weather data such as the amount of cloud and humidity. Then, it used the predicted solar radiation as input to a recursive neural network (RNN) that was trained for the month to forecast the PV output. Gensler et al. [35] combined an AutoEncoder (AE) and Long Short-Term Memory (LSTM), and trained it with the historical numerical weather prediction (NWP) data, and produced the power forecast in the 3-h resolution. The AE part tackled the feature extraction part, and the LSTM part captured the time-dependency of the model. The work showed that the hybrid model outperforms other models such as MLP, LSTM, and DBN. Grimaccia et al. [36] used the clear sky model and the historical weather forecast data for the day-ahead PV output forecast. It investigated the proper sizes of single hidden-layer and double hidden-layer feedforward networks. It concluded that a single hidden layer network with 120 neurons in the layer performs the best for their case. Ogliari et al. [37] also used the clear sky model with the weather forecast data. It combined a Social Network Optimization (SNO) technique with ANN, which was shown to perform better than either of them. Our work departs from these works in that we use a feedforward network with as many as four hidden layers with a large number (64) of neurons in each hidden layer. We aim to deal with the coarse granularity in the weather forecast data by adopting the large network size. In addition, unlike some of the above works that used RNN variants, we do not rely on the time dependency in the data.

## 3. Materials and Methods

In this section, we discuss the historical data that we use to train our DNN model. Then, we compare our model with the conventional system that relies on on-site sensors. We also discuss how we determine the input parameters and the hyperparameters for our DNN model.

### 3.1. Historical Data

We use two historical data sets, each accumulated over a period of a year. They are from the years 2014 and 2016, respectively. The data set from 2014 is composed of three parts:

- Weather forecast data posted every three hours by the Korean Meteorological Administration (KMA) with the forecast horizon of up to 67 h; collected during 7:00 a.m.–6:00 p.m. only
- On-site temperature, humidity, and solar radiation sensor measurement data from the installation (see Figure 1) in the same duration
- PV power output in the same duration

The KMA announces long/medium/short/very short-term weather forecasts. Among these, the 2014 data set is the accumulation of the short-term forecasts. The BEMS fetches this data from the KMA every three hours. The sensor data are from the Direct Digital Controller (DDC) connected to the sensors, which BEMS requests every five minutes. For our study, we averaged them into hourly data entries.

The 2016 data set is also composed of three parts:

- Hourly weather measurement data from the KMA
- On-site temperature, humidity, and solar radiation sensor measurement data from the installation in the same duration
- PV power output in the same duration

Note that, unlike the 2014 data, the 2016 data is not forecast data; it is actual measurements. We use this measurement data to determine the architecture of our DNN, such as the hyperparameters and the input parameters. After the architecture is determined, we apply some of the 2014 data to train the final forecast model that uses the remaining weather forecasts as test input. Table 1 summarizes the items in each data set.

#### 3.1.1. Weather Measurement Data from the KMA (2016)

**Figure 2:**
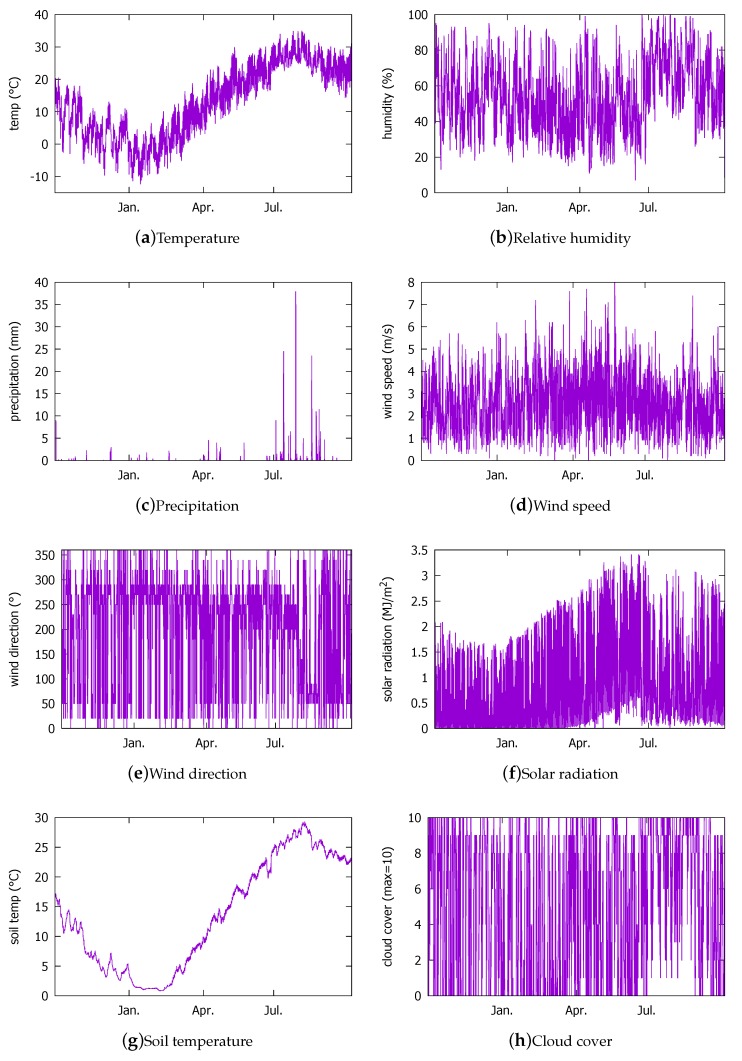
Hourly weather measurement data from the Korean Meteorological Administration (KMA) in year 2016 that are used in this study.

The first weather data set we use to determine the DNN architecture is the actual hourly measurement data provided by the KMA in 2016 [38]. The 2016 KMA data set contains the entries from 24 October 2016 through 26 September 2017. Thus, the data set roughly covers a whole year (i.e., all seasonal weather patterns). The weather measurements are for a region in the Seoul city where our solar power generation system is located (Latitude = 37.4702759${}^{\circ }$, Longitude = 126.8852906${}^{\circ }$). The weather data were collected from KMA only during the day because that is when the solar power generation can take place in our location. The data items are plotted in Figure 2. Note that the solar radiation (Figure 2f) has non-zero values mainly in summer months because of the data items only contain day hours. The solar radiation goes to zero during night, but, because of the day hour filtering, the zero values do not appear in the figure. From 11 June through 26 June and 29 July through 3 August, data are missing. During this period, there was a system shutdown following the irradiance sensor overcurrent that broke the DDC that led to its replacement and the reconfiguration of the BEMS. The lack of matching PV output values from the BEMS renders the corresponding weather measurement data unusable. As a result, we end up with 3798 entries at our disposal. Among the 3798 entries, we will use 3000 for training and 798 for validation.

#### 3.1.2. Measured Data from the On-Site Sensors (2016)

**Figure 3:**
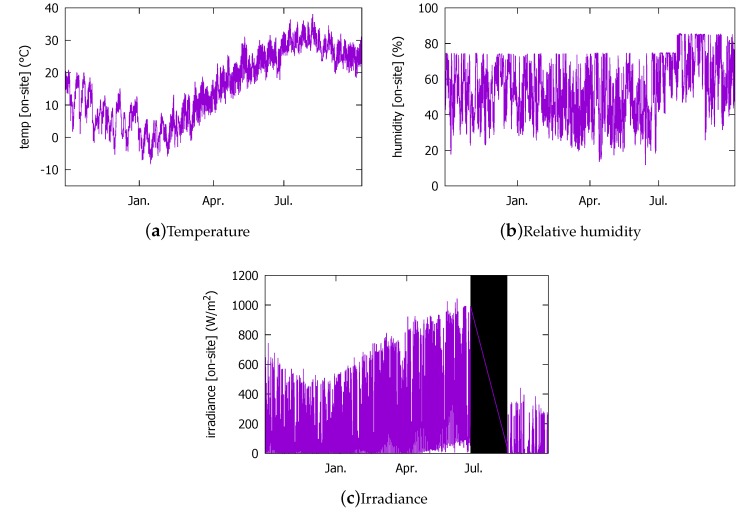
On-site sensory measurement data in the 2016 data set.

We measured the irradiance (W/m${}^{2}$), outdoor temperature (°C), and relative humidity (%) at the solar panels’ installation. They are not used by our sensorless approach; they are used to train the conventional forecast model. Among these, the irradiance is used as the target output value when we train the conventional model in Section 3.3. The installed solar panels can collectively generate up to 2.448 kW. The panels are 15${}^{\circ }$ tilted, facing South, and fixed on the roof of a commercial building in Seoul, Korea. Matching the weather measurement data, the sensory data were produced only during day. In addition to the system shutdown discussed above, the irradiance sensor data are not available from 11 June through 20 July. Thus, in Figure 3c, this period is blacked out. Moreover, the calibration has not been done satisfactorily following the irradiance sensor breakdown, so that the irradiance and the humidity sensors show erratic values after the shutdown period. In addition, the humidity sensor reports are quite different from the KMA data due to another calibration error. In fact, such difficulty of facility management is one of the reasons that we pursue an approach that does not depend on the on-site measurements’ data. The local calibration errors that are not as strictly filtered as in the typically more reliable weather data can render the model based on the on-site measurements imprecise. Lastly, as in Figure 2f, the irradiance values in Figure 3c in summer are above zero because only the day hour measurements are included in the data set.

**Figure 4:**
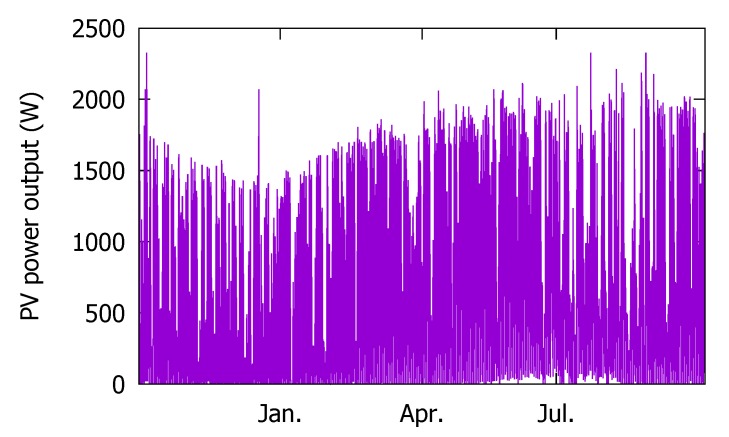
The 2016 photovoltaic (PV) power output data.

We also have the PV power output, as shown in Figure 4. We use it as the ground truth when training and validating our forecasting model based on the 2016 data set.

#### 3.1.3. Weather Forecast Data from the KMA (2014)

**Figure 5:**
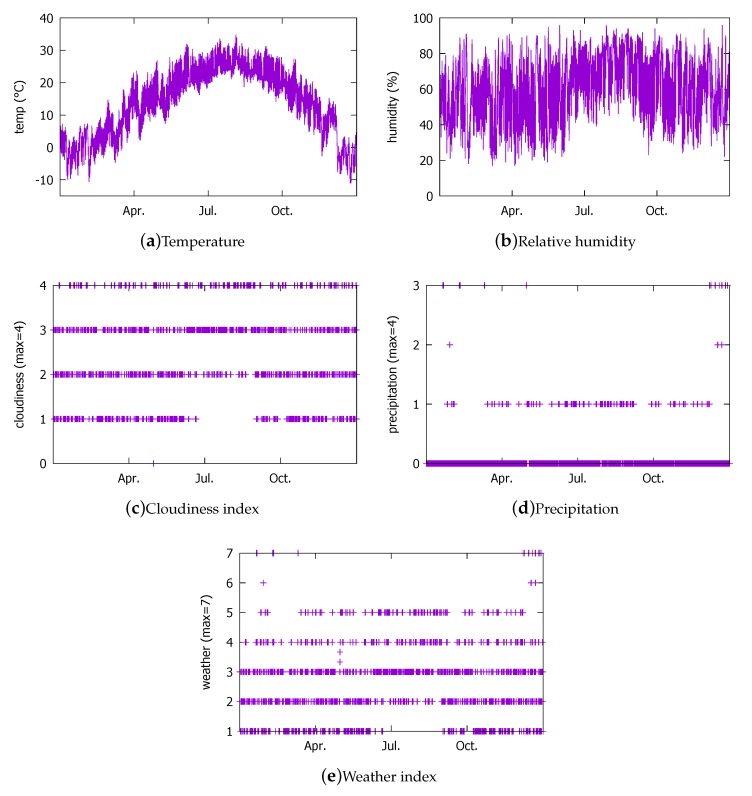
Some weather forecast data items from KMA in 2014.

The 2014 data set contains the KMA weather forecast data published every three hours for the horizons of 4 to 67 h in the geographical grid cell of a 5 km × 5 km size [38] where our facility is located. We collected them through the API that the KMA provides in the XML format, from 1 January through 31 December 2014. The total number of entries is 8410. The larger number of entries is because we did not filter the night hours as in 2016. This corresponds to 358 days, but here also we lost 182 entries due to system errors. Among the data items in the data set, we present some in Figure 5. Notice that the cloudiness, precipitation, and weather indices do not have corresponding data items in the 2016 measurement data. The precipitation index enumerates the type of precipitation (0: None, 1: Rain, 2: Rain/Snow, 3: Snow/Rain, 4: Snow). The cloudiness index roughly enumerates the cloud cover (1: Clear, 2: Partly Cloudy, 3: Mostly Cloudy, 4: Overcast). The weather index combines the cloudiness and the precipitation indices in the scale of 1 to 7 (1: Clear, 2: Partly Cloudy, 3: Mostly Cloudy, 4: Overcast, 5: Rain, 6: Rain/Snow, 7: Snow). We notice from the cloudiness index that in the rainy season, which starts in July, more clouds than clear skies are forecasted.

Since the weather forecast has the 3-h resolution, it cannot be directly matched with the actual PV power output that is generated more frequently. For the hourly PV power output forecast that we target, therefore, we linearly interpolate two intermediate points between two consecutive weather forecast entries to match the hourly PV output data. In future works, we will use a better interpolation to further reduce the prediction errors.

#### 3.1.4. Actual PV Power Output (2014)

**Figure 6:**
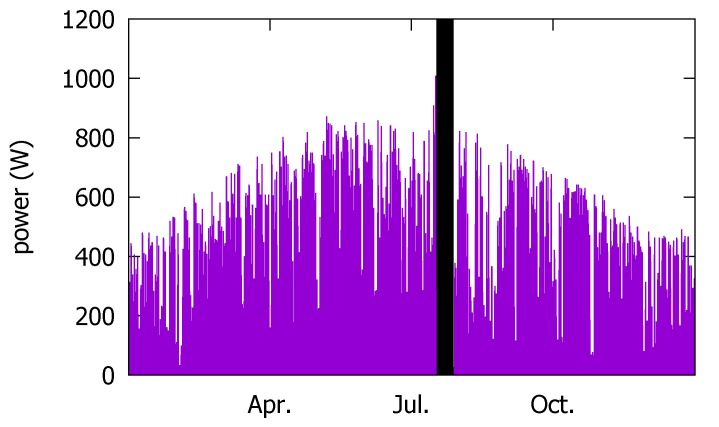
Actual PV power output in 2014.

Similar to 2016, we also collected the on-site sensor measurements. However, we do not use them to train our model. Thus, instead, we present the actual hourly PV power output Figure 6 as the ground truth to be used in training our forecast model. The PV power output measurement data are missing from 12 July through 22 July, due to the power conditioning system (PCS) device breakdown. The PV testbed installation specifications are given in Table 2. Notice that the capacity of the facility is half of that in 2016.

### 3.2. Comparison of the Existing and the Proposed Approaches

#### 3.2.1. Conventional Approach to PV Power Output Prediction

**Figure 7:**
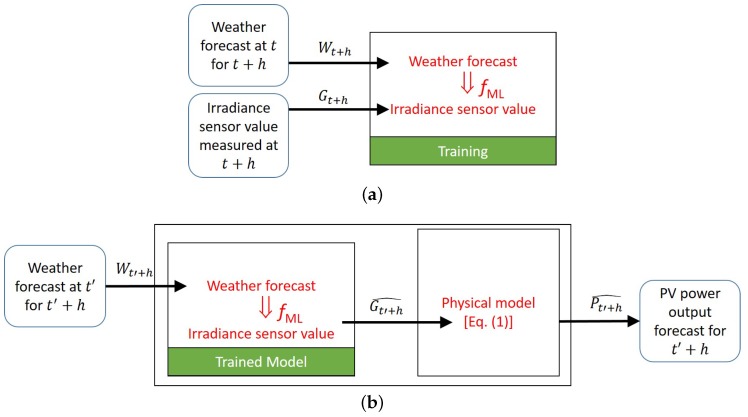
Conventional approach to forecasting PV power output: (**a**) training; (**b**); forecasting.

Typically, the PV power output forecasting takes a two-staged approach [3,35,39]. In the first stage, an NWP is created based on various techniques such as Autoregressive (AR) models, artificial neural networks (ANNs) [40], Fuzzy Logic, and hybrid systems [17,41]. The accumulated historical observations and meteorological data for the installation site are used to construct the regression model. In the second stage, based on this NWP, a forecasting algorithm typically implemented in commercial software predicts the future PV power output. In our existing system shown in Figure 1, we also run a two-staged forecast model depicted in Figure 7b. During training, the historical weather forecast $W_{t+h}$ made at *t* with the target horizon *h* and the corresponding on-site measured irradiance $G_{t+h}$ are used to train the irradiance forecast model $f_{ML}$ (Figure 7a). After training, the system uses the trained model at $t^{\prime }\gg t$ to forecast the irradiance $G_{t^{\prime }+h}=f_{ML}\left(W_{t^{\prime }+h}\right)$. For $f_{ML}$, we use the Gaussian Process regression model [42]. It predicts the irradiance to be the one that matches the most similar input values in the historical data. The inputs *W* are composed of the following:

- Expected angle of incidence of the Sun, considering the latitude, longitude, and panel tilting angle,
- Forecasted amount of precipitation,
- Forecasted cloudiness index,

whose relation with the measured irradiance at the forecasted time in the historical on-site data is modeled by $f_{ML}$. In the first stage of the forecasting at $t^{\prime }$, the system uses $f_{ML}$ and $W_{t^{\prime }+h}$ to predict $\hat{G_{t^{\prime }+h}}$.

Then, in the second stage, the conventional system uses $\hat{G_{t^{\prime }+h}}$ to compute the power $\hat{P_{t^{\prime }+h}}$ based on a physical model of the PV power output [43] as follows:

(1) $$\hat{P_{t^{\prime }+h}}=A_{surf}\cdot f_{activ}\cdot \hat{G_{t^{\prime }+h}}\cdot \eta_{cell}\cdot \eta_{invert},$$

where

- $A_{surf}$: net area of solar panel surface (m${}^{2}$),
- $f_{activ}$: fraction of surface area with active solar cell,
- $\eta_{cell}$: module conversion efficiency,
- $\eta_{invert}$: direct current (DC) to alternating current (AC) conversion efficiency.

Note here that $A_{surf}$ and $f_{activ}$ are fixed upon facility installation. In contrast, the irradiance is highly affected by the weather conditions. Although not as much, the conversion efficiency parameters ηs are also affected by the weather conditions because solar cell modules, made of semiconductors, are temperature-sensitive [44]. The ambient temperature, and the snow or sand dust particles that can cover the surface of the cell affect $\eta_{cell}$. The ambient temperature and the temperature inside the PCS device affect $\eta_{invert}$. Although ηs are functions of the environmental parameters, they are typically treated as constants based on the manufacturer specifications. However, because the site-specific situations such as the tilting angle and the shading can also affect them, they can become a source of prediction error.

An important aspect of the conventional approach is that the system should be accumulating the various sensor data at the site for the learning of $f_{ML}$. It means that we need to install the sensors in each facility site. It incurs the installation and operation costs [45,46]. In our case, for instance, the capital expenditure for the sensors and wiring, installation labor, and engineering total at $3000. In addition, the operation and maintenance costs keep being added to the overall cost. Furthermore, it can take a long time to accumulate a sufficient amount of data until we can use it to train the forecast model, another costly aspect of the conventional approach.

#### 3.2.2. Proposed Deep Learning Approach

**Figure 8:**
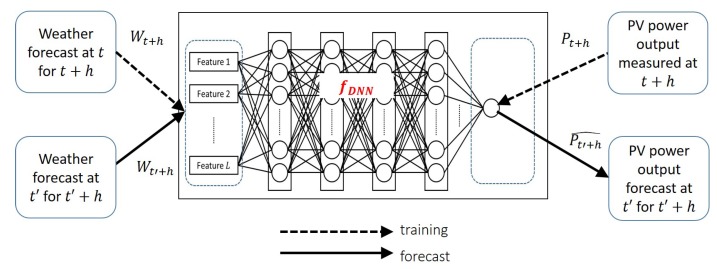
Deep Neural Network (DNN)-based model to forecast PV power output directly.

In order to tackle the aforementioned cost issues of sensor-involved forecasting, we explore a DNN-based sensorless alternative. The method is depicted in Figure 8. It directly trains the forecast model by using only the historical weather forecast data $W_{t+h}$ and the matching historical PV output $P_{t+h}$ as ground truth. After training, we use $f_{DNN}$ to obtain the forecast $\hat{P_{t^{\prime }+h}}=f_{DNN}\left(W_{t^{\prime }+h}\right)$ at $t^{\prime }\gg t$. Because the day-ahead forecast is of primary interest to grid-connected buildings, we focus on the horizon of h=24 h.

By deciding to omit the sensors in the prediction loop, however, we come to face the very issue that the sensor-based conventional approach solves through its first stage. Namely, we need to cope with the errors that are caused by the coarse granularity of the weather forecast. Temporally, the KMA publishes the forecast only every three hours. In quality, the amount of solar radiation is given in 1 to 4 scale (i.e., cloudiness index), instead of more precise value such as the irradiance [3] or even the cloud cover in 0 to 10 scale as in the KMA measurement data. However, an important premise in this paper is that the weather forecast errors are not completely random, and may have a complex pattern that we cannot easily model. Therefore, we believe that even that pattern can be captured by an appropriately sized DNN. In fact, we employ a large DNN that has not been used before in the PV power output forecasting literature. One caveat in using the large network is that insufficient training data leads to overfitting. However, we can overcome this hurdle by exploiting the weather forecasts from the past that are already accumulated over an extended period of time and readily available from public weather services. As long as the matching PV power output history exists in the target installation region with similar configuration parameters, we can immediately train the DNN model. Indeed, we demonstrate in the next section that we can achieve a higher level of prediction performance in almost all measures in this way without the support of on-site sensors.

**Figure 9:**
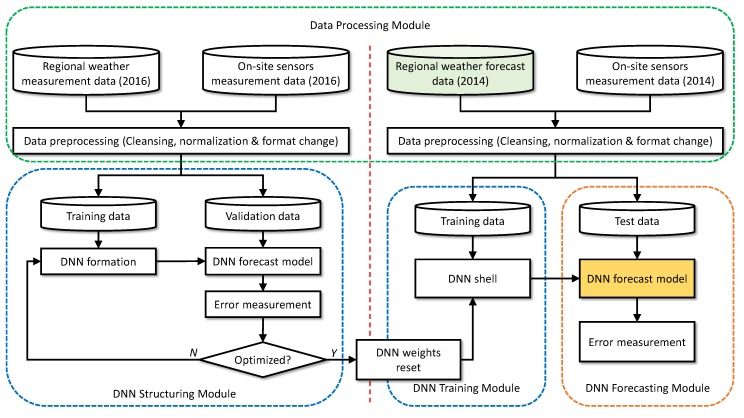
The procedure used in this paper.

Figure 9 summarizes how we proceed to determine the architecture of the DNN for $f_{DNN}$ and then train it. Note that we use the 2016 data set to determine the DNN architecture, and the 2014 data set to train the DNN for forecasting use. There are two reasons that we use the data sets for different purposes. First, the two data sets have different structures and contents as the 2016 data from the KMA are actual measurements, whereas the 2014 KMA data are forecasts (see Table 1). Second, the number of solar panels in 2016 in the system doubled from 2014, and so did the maximum power output (see Table 2). Due to these incompatibilities, we cannot directly combine the two years’ data to train a single model. However, the two data sets represent the fundamentally identical dynamics, so we believe that the DNN architecture that works best for the 2016 data set will be also good to capture the dynamics in the 2014 data set. Lastly, before using the data, we removed faulty data entries from the two data sets. We also eliminated the entries that only have either the weather data from the KMA or the sensory data from the installation. As for the peak power difference, we can circumvent the problem by normalizing the data, which also helps reduce the training time [31,35]. For instance, the PV power output in the 2014 data set was normalized with the maximum value of 1.224 kW mapped to 1. These normalized data are used for evaluation in Section 4.3 as well. For the final forecasting output, however, we reversed the normalization process so that absolute forecast values are obtained. Since the main focus of this paper is in comparing the DNN-based sensorless PV forecasting vs. sensor-assisted two-staged models, we do not apply more sophisticated data pre-processing techniques such as Wavelet Decomposition [25,28,47] to improve the forecast performance. However, we cannot emphasize the importance of the pre-processing too much, as it plays an essential role in the accurate forecasting.

The left half of Figure 9 illustrates the DNN architecturing based on the 2016 data set. It involves trying various hyperparameters and input parameters until we find the configuration that leads to the lowest average prediction error. For this trial-and-error process, we split the 2016 data entries into the training (3000) and the validation (798) subsets. Then, we reset the weights in the trained and validated DNN, leaving only the empty structure (“DNN shell” in the figure). On the right half of the figure, the 2014 data set is used to train this DNN. Here, we split the data set into the training (6000) and the test (2410) subsets.

### 3.3. Architecturing the DNN

#### 3.3.1. Selecting the Deep Learning Model

In the PV power output forecast literature, many previous works employ the long–short term memory (LSTM), Elman network, or the recursive neural network (RNN) in general. They are a popular DNN type to learn the time dependent nature of solar radiation [26,31,34,35,48,49,50]. However, considering the targeted hourly forecast under the much coarser time grain of 3 h in the weather forecast data, we decided not to rely on the time dependency between adjacent data entries. A positive side of sacrificing the time dependency is that the model will not be affected as much when the weather condition wildly varies between two forecasted hours. Without the time dependency between data entries, perhaps except through the time indices (month, date, and hour), we can choose a feedforward network for the DNN type. In this paper, we employ the Multi-Layer Perceptron (MLP). Note that our focus is not in proposing a DNN model having a better precision than others, or improving its learning speed. Rather, we aim to show that large DNNs can replace the traditional sensor-based approach by coping with coarse-grained weather forecast, whatever DNN type is selected.

**Figure 10:**
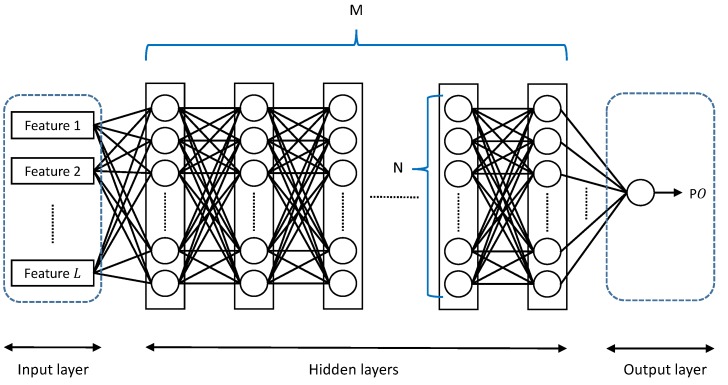
The Multilayer Perceptron (MLP) architecture.

**Figure 11:**
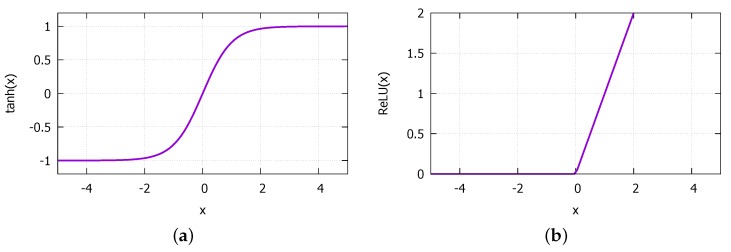
Activation functions: (**a**) hypertangent (tanh) used for hidden layers; (**b**) rectified Linear Unit (ReLU) activation function used for the output layer.

MLP is a neural network model that can approximate any nonlinear function (Figure 10). The MLP consists of an input layer that receives input parameters, an output layer that computes the modeled function value for the input. The hidden layers are where the learning takes place. Our MLP consists of *L* input parameters and *M* hidden layers each with *N* neurons that are fully connected between the adjacent layers. The input layer also has *N* neurons, and the output layer, only one. For the activation functions in the neural network, we employ the hypertangent (tanh) and the rectified linear unit (ReLU) that are shown in Figure 11. The former is used for all neurons in the hidden layers, and the latter, for the output layer. For the gradient descent optimization algorithm, we employ the Adaptive Moment Estimation (Adam) [51] that computes adaptive learning rates for each input parameter.

For learning the weights for the hidden neurons, the back propagation is done based on the loss function value. In this paper, we use the mean absolute error (MAE) for the loss function, defined as follows:

(2) $$MAE=\frac{1}{n}\sum_{i=1}^{n}\left|\hat{y_{i}}-y_{i}\right|,$$

where *n* is the number of data elements, $\hat{y_{i}}$ is the predicted PV power output, and $y_{i}$ the ground truth. Recollect that we normalized the data items by their minimum and maximum values before we train the models (see Figure 9). Specifically, all values of $y_{i}$, $\hat{y_{i}}$, $\bar{y_{i}}$ and $\bar{\hat{y_{i}}}$ have values between 0 and 1.

#### 3.3.2. Searching for Appropriate Hyperparameters and Input Parameters

The learning process using the MLP is affected by the number of hidden layers (*M*) and neurons therein (*N*), training data batch size (*B*), number of epochs (*E*), and the input parameter combination, etc. Therefore, we need to carefully determine these parameters for better prediction performance. In this paper, we use the trial-and-error method to find the appropriate architecture. For the initial configuration, we try M=1, N=64, and E=1000. In order to not explode the search space, we fix *N* and *B* at 64 and 360, respectively. In addition, we use α=0.001 for the learning rate for all combinations. We explore the values of *M*, *E*, and *L* below. As the initial configuration, we set M=1, E=1000, and for the input parameters we start with all input features but the on-site measured irradiance from the year 2016 (L=13). We exclude the irradiance as possible input because the final DNN model in Section 4 uses only the weather forecast as input, where the irradiance is not included.

**Figure 12:**
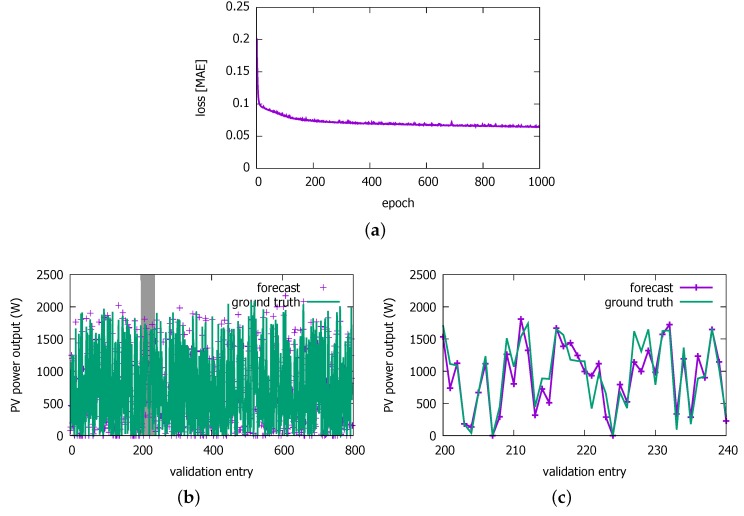
A PV power output learning result for 2016 data set. (**a**) loss against training data set; (**b**) prediction results against validation data set; (**c**) detailed prediction results on validation data set (shaded band in (**b**)).

Figure 12a shows the loss value as a function of the epochs. We observe that the loss gradually decreases until E=1000. Then, we apply this trained model to predicting the PV power output for the entries in the validation data set. Figure 12b and c show the result. We see that the prediction roughly matches with the power output values in the validation data set. Note that the validation entries are not in the chronological order, as we put randomly selected entries in each batch in both training and validation.

For the subsequent explorations with different parameter combinations, we repeat the same process of measuring the MAE loss function value of the given configuration.

#### 3.3.3. Training Time *E*

**Figure 13:**
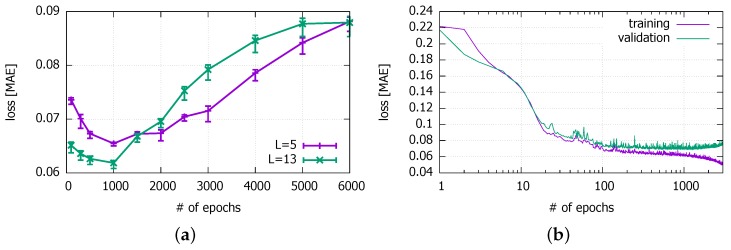
Impact of epochs on errors. (**a**) by trial-and-error (with 95% confidence interval); (**b**) by identifying where the performance against the validation data set begins to worsen—the early stopping criterion (for L=5).

Next, we try various numbers of epochs *E* from 100 up to 6000 to find the optimal training time. Figure 13a shows the results with two different values of *L*. We find that E=1000 happens to have the lowest MAE for the initial configuration. This number of the appropriate training epochs of our model is much larger than most other works [24,31,48,49,52]. We also confirm in the figure that using more features can achieve the lower minimum MAE, as found in earlier works [3]. With a smaller number of input features L=5 as chosen in Section 3.3.5, the optimal number of training epochs is 1000 as well. Thus, in the subsequent experiments and in the next section, we use E=1000. Another interesting observation in the figure is that, for E<1000, a larger number of input features lead to smaller losses, whereas it leads to larger losses for E>1000. It is evidence that the DNN is overfitted faster with the larger number of input features beyond the optimal point of training. We will need more training data with L=13 to better cope with the overfitting.

In order to determine the precise number of epochs to avoid overfitting, we can more extensively search the epoch space and use the early stopping criterion [53]. In Figure 13b, we can find the point where the loss value begins to increase for the validation data set. The MAE for the validation set is stable (i.e., almost no change) between 500 and 1200 epochs, but starts to increase beyond 1200 epochs. The minimum loss occurs at E=1081. However, the MAE value there is similar to what we achieve with the 1000 epochs that we decided on with the trial-and-error method.

#### 3.3.4. Number of Hidden Layers *M*

Here, we try varying the number of hidden layers to from M=1 to M=6 with E=1000 and L=13. We summarize the results in Table 3. Since M=3 and M=4 are the configurations that produce the two lowest losses at E=1000, we further explore different numbers of epochs around them, but E=1000 and M=4 still performs the best. Thus, we use these values in the subsequent discussions.

Note that the network with four hidden layers with 64 neurons per layer is the largest DNN in the PV power output forecasting literature. The fact that the lowest loss is achieved with four layers but not one implies that using all 13 features in the weather measurements requires a complex model to capture their impacts on the PV power output. Worse yet, we have to cope with a more coarse forecast data in time granularity and in quality in Section 4. For this reason, we will keep the number of layers at four in the subsequent discussions although we will try to narrow down the list of input features to use below.

#### 3.3.5. Input Features *L*

Now, we explore the input parameter space, and we try to select the most relevant ones from the original 13. Incorporating too many input parameters in a DNN can bring about several issues. First, it increases the training time, particularly for the MLP network with full connectivity among neurons. Second, it increases the number of local optima in the error function, resulting in higher risks of suboptimal convergence [54]. Finally, by adding more dimensions, they require a bigger data set to populate the parameter space densely enough to represent an accurate mapping relationship [55]. With the minimally redundant data, however, the computation complexity becomes more sustainable, and leads to higher forecast accuracy. This is why prior studies strive to pick out the most influential meteorological parameters [56,57,58].

Among the 13 input features in the 2016 data set in Table 1, we select five as per the following judgements:

- Exclude the on-site temperature and humidity sensor values in addition to the already excluded irradiance because there are no matching values available in the 2014 weather forecast data.
- Keep the cloud cover. Although it does not have an identical item in the 2014 data set, we can approximate it with the cloudiness index there.
- Exclude the precipitation because there is strong dependency between the precipitation and the relative humidity (KMA). In other words, the relative humidity is always 100 % when it rains. Thus, pick the relative humidity instead.
- Exclude the soil temperature because it is the least relevant.
- Keep the month and the hour because the season and the time-of-day directly affect the solar radiation. However, exclude the date that is less likely to have correlation with the solar radiation or other weather conditions.

For other input features, we consider their correlation with the PV power output as shown in Table 4. Due to the low cross-correlation, the wind speed and the direction are discarded.

Table 5 shows the MAE values for different combinations of input parameters used in the 2016 model. As expected, the best result is obtained when all the climate inputs were applied to the system. However, the 5-parameter combination that we decided on comes second, better than other combinations with more input features. The other combinations that look to have the same MAE are slightly larger in the lower decimal places. Finally, it is worthwhile to note that one can apply more advanced techniques such as Principal Component Analysis (PCA) [47] to remove redundancy in the historical data more systematically.

To summarize this section, we decided on M=4, N=64, E=1000, and B=360 for the hyperparameters. For the input parameters, we use the following five:

- Month,
- Hour,
- Cloud cover,
- Temperature,
- Relative humidity.

## 4. Results

In this section, we inherit the DNN architecture determined in the previous section, and train the PV power output model using only the KMA weather forecast data from 2014. The training process itself is identical, so we omit the lengthy discussion on it. However, there are two remarks on the input and the output, respectively, from which we start our discussion on the final results.

### 4.1. Changes in Input and Output

There is one important change in the five input features we decided on in the previous section. The cloud cover does not have a direct equivalent in the 2014 data set (see Table 1). Instead, we have the cloudiness index in the weather forecast, so we substitute the index for the cloud cover. Then, the weather forecast has two items that contain the cloudiness information: the cloudiness index and the weather index. The latter is actually a combination of the cloudiness index and the precipitation index, and has seven values (1: Clear, 2: Partly Cloudy, 3: Mostly Cloudy, 4: Overcast, 5: Rain, 6: Rain/Snow, 7: Snow) as we discussed in Section 3.1.3. In this paper, we decided to use the combined weather index to replace the cloud cover as it contains more information. As for the output, unlike the solar power generation system in 2016 that could generate up to 2.448 kW, the 2014 system had 1.224 kW as the maximum power that solar panels could generate. The actual generate power in 2014 ranged from 0 to 1.008 kW, and we use it as ground truth for training.

### 4.2. Visual Comparison

**Figure 14:**
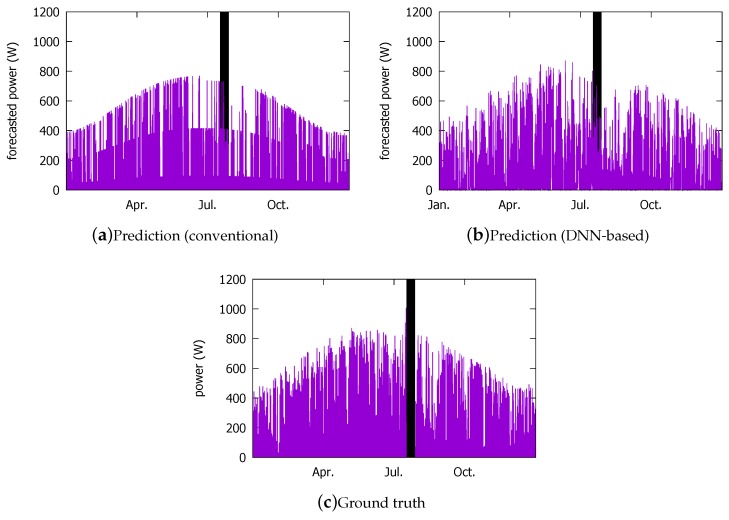
Visual comparison: (**a**) forecast from the current system; (**b**) DNN forecast (for test entries); (**c**) ground truth.

Below, we compare the prediction performance of the DNN model with that of the conventional approach that we have been using for our current system shown in Figure 1 in detail. However, first, we visually summarize the prediction performance in Figure 14. Comparing with the ground truth shown in Figure 14c, we observe that the DNN model (Figure 14b) is visually more similar than the conventional method (Figure 14a).

**Figure 15:**
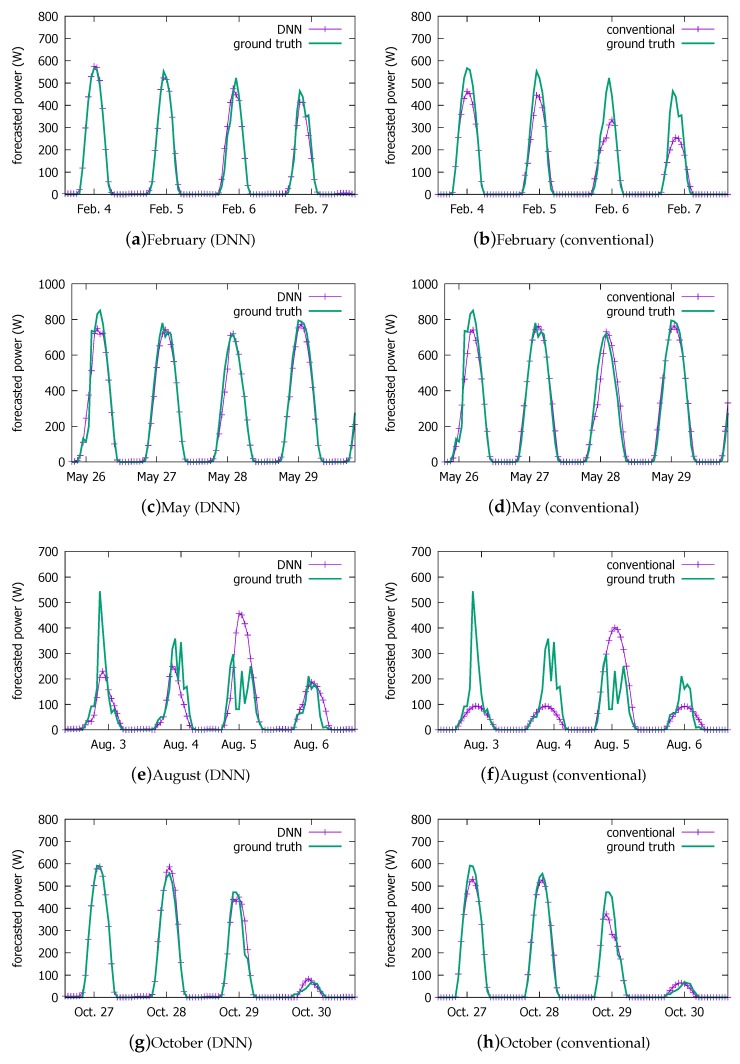
Forecast performance comparison against ground truth on some days over a year: the DNN model on the left column and the current system on the right.

Figure 15 magnifies a few days from the above forecasts by the two schemes, and puts them against the ground truth. For readability, we plot their predictions for approximately four days in each season. We list the weather and its changes that are forecasted by the KMA in Table 6. When the weather is bad, the prediction becomes more difficult for both models. In particular, the summer days (e and f) pose the greatest challenge due to the Monsoon climate. The current system tends to more severely underestimate the PV power output under bad weather conditions (6 and 7 February, and 3, 4, 6 August ). However, the DNN model more closely follows the peaks than the conventional model, except on 5 August when it overestimates slightly more than the current system.

### 4.3. Numeric Comparison

Having visually confirmed the better performance of the DNN model above, we compare the two schemes more quantitatively here. To do so, we begin by listing several widely used metrics in the literature that we will employ to measure the model performance over the entire test data set from 2016.

#### 4.3.1. Performance Measures

In this paper, we use five different metrics [35], Root Mean Square Error (RMSE), Mean Absolute Error (MAE), Absolute Deviation (AbsDev), Bias, and Correlation, as defined below. RMSE and MAE are strongly correlated with each other [37], but together they provide an insight on the forecast performance. If RMSE ≫ MAE, it means that the forecast has high deviations to the measured power output. If RMSE ≈ MAE, on the other hand, the forecast has only small deviations to the measured power output. The Bias allows for assessing whether power forecast is predicting higher or lower values than the measured power output. The AbsDev measure is commonly used within the energy sector to assess the quality of the forecast. The Correlation is an additional measure to assess the similarity of the power forecast and the measured power output:

$$\begin{array}{ccc} RMSE & = & \sqrt{\frac{1}{n}\cdot \sum_{i=1}^{n}{\left(\hat{y_{i}}-y_{i}\right)}^{2}},\\ MAE & = & \frac{1}{n}\cdot \sum_{i=1}^{n}\left(\hat{y_{i}}-y_{i}\right),\\ AbsDev & = & \frac{\sum_{i=1}^{n}\left(\hat{y_{i}}-y_{i}\right)}{\sum_{i=1}^{n}\;y_{i}},\\ Bias & = & \frac{1}{n}\cdot \sum_{i=1}^{n}\left(\hat{y_{i}}-y_{i}\right),\\ Corr & = & \frac{\sum_{i=1}^{n}\left(y_{i}-\bar{y_{i}}\right)\cdot \sum_{i=1}^{n}\left(\hat{y_{i}}-\bar{\hat{y_{i}}}\right)}{\sqrt{\sum_{i=1}^{n}{\left(y_{i}-\bar{y_{i}}\right)}^{2}\cdot \sum_{i=1}^{n}{\left(\hat{y_{i}}-\bar{\hat{y_{i}}}\right)}^{2}}}. \end{array}$$

#### 4.3.2. Comparison with the Conventional System

Table 7 shows the numeric performance comparison. The numbers are normalized, so 1.224 kW should be multiplied to obtain the corresponding absolute values. The differences between RMSE and MAE in the two schemes are comparable, and, since RMSE ≫ MAE, we can see that both forecasts have high deviations to the measured power output [35]. The Bias shows that the DNN model forecast is slightly overshooting, whereas the current system is predicting lower values than the measured power output. The Correlation tells us that the similarity of the DNN forecast and the measured power output is higher than the current system. Based on this result, we argue that the deep learning model can replace the conventional forecast method that utilizes on-site sensor measurements. From AbsDev, we observe that the quality of the forecast using the DNN model is considered better than the current system. Finally, we also observe that the quality of our feedforward forecast model is no worse than other works that explicitly exploit the time-dependency through recursive networks [35].

#### 4.3.3. Seosonal Performance of the DNN-Based Forecast Model

Now, we shed light on two detailed aspects of the DNN prediction performance above. First, we look at its seasonal performance. Table 8 shows the five measures for each season. Here, we only show the performance for the test data set.

We can observe that the DNN model suffers the worst performance in the summer. The correlations are comparable among Spring, Autumn, and Winter, but it is visibly lower in Summer. AbsDev also shows that the quality of the forecast is the lowest in the season. The largest variability we saw in Figure 15 is also corroborated by the largest difference between RMSE and MAE. The poor performance in Summer is due to the volatile weather conditions for the East-Asia Monsoon climate that we have in Korean peninsula [59]. The precipitation in the peninsula is concentrated in Summer, when 50% of the annual precipitation falls from June through August. The rainy season starts in late June, and the tropical storms frequent the region from June through October. Most regions in the peninsula receive more than 1000 mm of precipitation. Therefore, in a future work, we need to develop a more sophisticated model for the summer season to improve the precision, although a large part of the precision will still depend on the accuracy of the weather forecast, or, we could run multiple networks each of which applies to a particular weather type, as in Chen et al. [3]. In contrast, the deviation indicated by the difference of RMSE and MAE is the smallest in winter. It is because there are more clear days in the season than others as the Korean Peninsula enters the cold and dry Siberian high-pressure zone. The winter precipitation is less than 10% of the total annual precipitation [59].

#### 4.3.4. Performance of the DNN-Based Forecast Model under Different Types of Weather

Next, we look at the DNN model performance in different daily forecasted weather conditions. Recollect that the weather index incorporates the cloudiness index. Thus, Table 9 can be considered to show the performance under different levels of cloudiness. As we can expect, the best performance is obtained under the clear weather. From the correlation, we confirm that the DNN model has the greatest error for the Overcast day, followed by the Rainy/Snowy day. The AbsDev shows that the forecast quality follows the same order. The RMSE-MAE differences in the bad weather conditions reveal that the forecasts deviate significantly from the measured power output. The Cloudy is where the highest deviation is, followed by the Overcast and the Rain/Snow. Again, our future work will have to focus on these bad weather conditions for the overall improvement of the proposed approach.

#### 4.3.5. Comparison with an ANN-Based Model

As for the DNN dimensioning, it is worthwhile to re-evaluate our decision to use DNN instead of a single hidden-layer ANN. For this purpose, we finally consider two ANNs with a single hidden layer, which have N=10 and N=120 neurons in the hidden layer, respectively. The former we consider because most ANN works use a small number of neurons for the hidden layer, around 10. As for the latter, we blindly draw the number from a recent work [36]. Using the same five input features that we employ for our final DNN model and the same training and validation procedure, Table 10 compares the performances. We observe that the DNN-based model outperforms both ANN models with different *M* and *N* values in our case, except in Bias. In fact, the performance of the ANN models is no better than the conventional system shown in Table 7 except in terms of Correlation. The result reaffirms that the DNN model is a relatively good model for our data, and our decision to use the DNN-based approach paid off. Nevertheless, it cannot be generalized as the comparison of DNN vs. ANN, as the choice of numerous hyperparameters, input features, activation functions, layout, and, most importantly, the data set used for training among others can all affect the performance.

## 5. Discussion

Although we showed that the sensorless forecast using a large DNN has a better prediction quality than the two-stage model relying on on-site sensors, the prediction performances during Summer and under cloudy weather conditions are not satisfactory. From the perspective of the building energy management system, it badly needs the assistance of the forecast system when the weather is most unpredictable. To improve the forecast quality in these adverse conditions, we can consider several future directions of exploration. First, we can use more weather forecast data and the corresponding PV output data from the past years (e.g., year 2017 as well) to expose our model to more weather patterns. Second, we can additionally consider the time dependency of the weather data. Note that we completely ignored the time dependency in the current study. We believe that the time-dependent changes around the entry points that lead to the largest errors may be useful to reduce the errors.

In the current work, we did not explore a few dimensions of the DNN architecture. In particular, we fixed the number of neurons *N* in the hidden layer. However, some recent works [36,37] spearheaded the investigation on the hidden layer dimensioning. When we come to use more training data as discussed above, the DNN dimensioning parameters that we used in this paper may become irrelevant. In our future work, we will also explore the number of neurons in each hidden layer. Since it can be a time-consuming process, we will have to use more systematic techniques, in particular, the early stopping criterion [53].

## 6. Conclusions

The main lesson obtained through this study is that solving the weather data granularity and quality problem in PV output power forecasting does not have a single solution, namely fine-tuning it with local measurements using on-site sensors. We demonstrate that deep neural networks (DNNs) can achieve comparable or even slightly better forecast quality than our conventional two-stage system that relies on the on-site sensors, by training it with our PV power output history and the corresponding regional weather forecast data from the national weather service for one year. In essence, this finding tells us that most part of the PV power forecat system can be converted to software, by obviating the need of hardware (sensor) modules and their management. It will contribute to the cost, complexity, and reliability aspects of the energy management system in grid-connected buildings. We believe that the DNN-based forecast model can simplify the grid-connected building energy management systems (BEMS), making it more attractive in future.

## Figures and Tables

**Table 1:** Historical data sets with their features as potential input parameters to our forecast model (KMA: Korean Meteorological Administration).

2016 (Hourly Measurements)	Source	2014 (Forecast every 3 h up to 67 h Horizon)	Source
Month		Month	
Date		Date	
Hour		Hour	
Temperature (${}^{\circ }$C)	KMA	Temperature (${}^{\circ }$C)	KMA
-		Daily high temp. (${}^{\circ }$C)	KMA
-		Daily low temp. (${}^{\circ }$C)	KMA
Precipitation (mm)	KMA	Amount of rain (mm; next 12 h)	KMA
-		Amount of snow (mm; next 12 h)	KMA
-		Prob. of precipitation (%)	KMA
Wind speed (m/s)	KMA	Wind speed (m/s)	KMA
Wind direction (0–36; in 10${}^{\circ }$)	KMA	Wind direction (0–36; in 10${}^{\circ }$)	KMA
Relative humidity (%)	KMA	Relative humidity (%)	KMA
Solar radiation (MJ/m${}^{2}$)	KMA	-	
Cloud cover (0–10)	KMA	-	
Soil temp. (${}^{\circ }$C; 30 cm below surface)	KMA	-	
-		Cloudiness index	KMA
-		Precipitation index	KMA
-		Weather index	KMA
Temperature (${}^{\circ }$C)	On-site sensor	-	
Relative humidity (%)	On-site sensor	-	
Irradiance (W/m${}^{2}$)	On-site sensor	-	

**Table 2:** Photovoltaic (PV) testbed specification.

Parameter	Value
Longitude	126.8852906${}^{\circ }$E
Latitude	37.4702759${}^{\circ }$N
Altitude	68 m
Azimuth	180${}^{\circ }$
Tilt	15${}^{\circ }$
Mounting disposition	Flat roof
Field type	Fixed tilted plane
Installed capacity	1.224 kWp (2014)/2.448 kWp (2016)
Technology	Amorphous silicon
PV module	Solar Laminate PVL-136

**Table 3:** Prediction performance of some configurations, L=13 (MAE: Mean Absolute Error).

M	E	MAE	E	MAE
1	1000	0.067		
2	1000	0.066		
3			500	0.066
	1000	0.064		
			1500	0.064
			2000	0.068
4			500	0.065
	1000	0.062		
			1500	0.068
			2000	0.070
5	1000	0.067		
6	1000	0.067		

**Table 4:** Pearson product-moment correlation coefficient between input features and PV power output.

Weather Condition	Pearson Cross-Correlation Coefficient				
	Cloud Cover	Temperature	Humidity	Wind Speed	Wind Direction
Clear	0.000	0.175	−0.062	−0.044	−0.122
Cloudy	−0.104	0.219	−0.178	0.058	−0.086
Overcast	−0.015	0.259	−0.104	0.002	0.000
Rainy	−0.222	0.226	−0.189	−0.095	−0.027
Total	−0.352	0.129	−0.287	0.032	0.035

**Table 5:** Impacts of features on loss.

L	Excluded Features from Table 1	MAE
13	Irradiance	0.062
9	(13)+**Other** on-site sensor features, date	0.069
8	(9)+Soil temperature	0.071
7	(8)+Precipitation	0.067
6	(7)+Wind speed	0.067
6	(7)+Wind direction	0.070
5	(8)+Wind speed/direction	0.067

**Table 6:** Weather Forecast from KMA on the Exemplified Days.

Month	Date	Forecasted Weather Changes during Day Hours (7:00 a.m.–6:00 p.m.)
February	4	Clear
	5	Partly Cloudy
	6	Mostly Cloudy → Partly Cloudy
	7	Partly Cloudy → Mostly Cloudy → Overcast
May	26	Mostly Cloudy → Partly Cloudy → Clear
	27	Clear
	28	Mostly Cloudy → Partly Cloudy
	29	Clear → Partly Cloudy
August	3	Rain
	4	Overcast → Rain
	5	Mostly Cloudy
	6	Overcast → Rain → Overcast
October	27	Clear
	28	Clear
	29	Partly Cloudy
	30	Partly Cloudy → Mostly Cloudy

**Table 7:** Forecast performance in five metrics.

	DNN Model		Current System	
	Training	Test	Training	Test
RMSE	0.061	0.064	0.071	0.066
MAE	0.027	0.029	0.033	0.030
AbsDev	0.239	0.266	0.291	0.277
Bias	0.007	0.008	−0.004	−0.002
Correlation	0.936	0.929	0.910	0.921

**Table 8:** Seasonal forecast performance of the DNN model.

	Spring (March–May)	Summer (June–August)	Autumn (September–November)	Winter (December–February)
RMSE	0.057	0.100	0.045	0.032
MAE	0.028	0.052	0.020	0.015
AbsDev	0.195	0.477	0.190	0.208
Bias	0.009	0.010	0.008	0.004
Correlation	0.962	0.819	0.965	0.966

**Table 9:** Forecast performance in different weather indices.

	Clear (1)	Cloudy (2 to 3)	Overcast (4)	Rain/Snow (5 to 7)
RMSE	0.026	0.051	0.059	0.051
MAE	0.012	0.018	0.027	0.023
AbsDev	0.107	0.227	0.600	0.484
Bias	0.006	0.004	0.012	−0.005
Correlation	0.991	0.935	0.778	0.833

**Table 10:** Comparison with ANN models with a single hidden layer.

	DNN Model	ANN Model (*N* = 10)	ANN Model (*N* = 120)
RMSE	0.064	0.068	0.068
MAE	0.029	0.034	0.034
AbsDev	0.266	0.310	0.320
Bias	0.008	0.004	0.005
Correlation	0.929	0.916	0.915

## Supplementary Materials

The 2014 and 2016 KMA data sets are available online at https://widen.korea.ac.kr/wiki/index.php/KU-KMAdata.
